# The Psychological Nature of Female Gait Attractiveness

**DOI:** 10.3390/bioengineering11101037

**Published:** 2024-10-17

**Authors:** Hiroko Tanabe, Kota Yamamoto

**Affiliations:** 1 Graduate School of Humanities and Human Sciences, Hokkaido University, Kita 10, Nishi 7, Kita-ku, Sapporo 060-0810, Japan; 2 School of Humanities, Hokusei Gakuen University, 2-3-1, Ohyachi-Nishi, Atsubetsu-ku, Sapporo 004-8631, Japan; ko-yamamoto@hokusei.ac.jp

**Keywords:** gait, attractiveness, self-expression, interpersonal cognition, nonverbal communication, structural equation modeling, gait observation, gaze behavior

## Abstract

Walking, a basic physical movement of the human body, is a resource for observers in forming interpersonal impressions. We have previously investigated the expression and perception of the attractiveness of female gaits. In this paper, drawing on our previous research, additional analysis, and reviewing previous studies, we seek to deepen our understanding of the function of gait attractiveness. First, we review previous research on gait as nonverbal information. Then, we show that fashion models’ gaits reflect sociocultural genderlessness, while nonmodels express reproductive-related biological attractiveness. Next, we discuss the functions of gait attractiveness based on statistical models that link gait parameters and attractiveness scores. Finally, we focus on observers’ perception of attractiveness, constructing a model of the visual information processing with respect to gait attractiveness. Overall, our results suggest that there are not only biological but also sociocultural criteria for gait attractiveness, and men and women place greater importance on the former and latter criteria, respectively, when assessing female gait attractiveness. This paper forms a major step forward in neuroaesthetics to understand the beauty of the human body and the generation of biological motions.

## 1. Introduction

The movements of the human body interact with the social environment. In other words, human movements can be modulated by social stimuli and can convey nonverbal messages, taking the form of social stimuli, such as the actor’s interpersonal attitude, intention, gender, and emotional state (e.g., inclusive or noninclusive attitudes toward a person that are implicitly conveyed by the physical positioning of two people) [1,2,3,4,5]. Charles Darwin systematically described the connection between emotions and behavioral expression, clarifying the role of animals’ bodily expression in their social environment [6]. Darwin’s theory was developed into the Laban movement analysis, which describes emotional expressions, such as the characteristics and quality of movement (effort) and geometrical characteristics (shape) [7,8]. In addition, Norton classified linguistic and paralinguistic information (including body motion) into nine communicative styles (i.e., dramatic, dominant, animated, relaxed, attentive, open, friendly, argumentative, and impression-leaving) in relation to how observers interpret and give meaning to it [9]. These styles have three dimensions, assertiveness, responsiveness, and versatility, relating to the ability to attract or control the observer’s interest and attention, his or her emotional, flexible, and approachable nature, and the ability to change behavior in response to others, respectively [10]. In this way, human body movements have an important role to play in interpersonal communication, conveying the psychological state of the person who is performing the movement.

Gait, the most basic human movement, functions as a social stimulus, conveying the attributes and psychological state of the walker as nonverbal information. Among other features, there are gender differences in gait kinematics, reflected in hip flexion angle, knee flexion moment, and pelvic tilt angle [11,12,13]. Using these differences, it is possible to generate motions of gait that are characteristic of each gender [14]. Age also alters gait, including increases in step width and double support period and decreases in stride length, cadence, gait symmetry, toe-off angle, and walking speed [15,16]. In addition, gait reflects the walker’s emotions, and observers can estimate a walker’s emotions from walking speed, posture, and dynamic cues [17,18,19,20,21]. Even where interpersonal communication is active, gait patterns affect the evaluations made by others. For example, in job interviews, the gait patterns of applicants convey an impression of their temperament and personality [22]. In this way, walking motion embodies the physiological and psychological states of the walker. However, it remains unclear what specific dynamic cues in gait affect interpersonal communication and what functions they have.

Immediacy, defined as a certain level of physical and psychological intimacy that is recognized between two people [10], is important for smooth interpersonal communication. People who behave in a way characterized by high immediacy are more likely to be liked by others, highly evaluated, and selected [5,23]. In nonverbal immediacy, physical attractiveness in appearance and behavior plays an important role in making others perceive one as attractive, thus increasing the closeness between two people [10]. Many previous studies have identified the characteristics of attractive physical appearance. For example, it has been reported that men who have a muscular body shape, called mesomorphic [24,25,26], and male bodies with healthy amounts of fat and muscle mass [27,28] are considered attractive by women. In addition, waist-to-hip ratio [29] and lumbar curvature, when viewed from the side [30,31], are cues for the evaluation of attractiveness in female bodies. Because these physical characteristics are related to health and reproductive function, physical attractiveness may function as material for detecting individuals’ health [32,33]. This is the dominant evolutionary psychological interpretation of the function of physical attractiveness. However, in some cases, physical features deviating from a healthy state are evaluated as attractive, especially in female bodies (e.g., a BMI [28] and fat mass [34] lower than the healthy level are considered attractive), which may be influenced by the cultural environment reflected by mass media [35]. To the best of our knowledge, there is no consistent view of physical attractiveness, including in the context of the sociocultural environment. In addition, physical attractiveness is not only seen in static representations of the face and body but also in physical movements (dance, walking, gestures, etc. [36,37]), and Morris et al. suggested that stride length and hip rotation are factors in the assessment in the attractiveness of the female gait [38]. However, detailed whole-body features related to attractiveness have not been elucidated, and the causal relationship between the parameters of the gait and impression evaluation, as well as how observers perceive the attractiveness of gait, remain unclear. Against this background, we have conducted research into the expression of attractiveness in terms of walking movements and the perception and evaluation of gait attractiveness [39,40,41].

In psychology, “Attractiveness refers to a person’s perceived qualities, characteristics, or traits that evoke positive feelings, interest, or desire for social interaction. The multifaceted concept of attractiveness encompasses several components, including physical appearance, personality, intelligence, social skills, and other traits.” [42]. In this study, we focus on attractiveness as perceived by observers in terms of physical appearance, including human movement. Following our previous research on the expression and perception of gait attractiveness and using additional data analysis, we seek to deepen our understanding of the function of gait attractiveness, as well as construct a model of the visual information processing process in gait attractiveness perception. Along with reviews of various previous studies on nonverbal communication, evolutionary psychology, and visual information processing, we structured this paper in the following three sections.

- Section 2: Expressing the attractiveness of female walkers. What gait patterns do women use to express attractiveness and increase their immediacy to others? From our previous work [39] and additional analyses of comparisons between fashion models and nonmodels, as well as impression evaluation by observers, we comprehensively identify the expression of physical attractiveness in walking motion.
- Section 3: Attractiveness evaluation model of female gait. We outline statistical estimation models for female gait attractiveness using the parameters of gait as explanatory variables [40]. The models we constructed partially show elements that may be inconsistent with evolutionary adaptations. Thus, we discuss the influence of the biological and sociocultural environment on the attractiveness of physical movements in relation to a review of previous research.
- Section 4: Perception of gait attractiveness. From our research on gaze behavior and its gender differences when evaluating gait attractiveness [41], we discuss how the visual gait information related to attractiveness is processed in observers, conducting a review of previous research. Finally, we develop a neuroaesthetic model for visual information processing for gait attractiveness.

In this paper, we use the terms “gender” and “sex” differently depending on how previous research is described. Moreover, in our data analysis and interpretation, we use the term “sex” instead of “gender” because the latter includes the meaning of self-representation of one’s own sexuality, which is not covered in this study.

## 2. Expressing the Attractiveness of Female Walkers

### 2.1. Biomechanical Strategies to Maximize Gait Attractiveness

Nonverbal behavior has the function of increasing immediacy in interpersonal relationships. Gait kinematics may be a cue for female attractiveness, which is a factor in increasing immediacy [43]. Gait can also exhibit the intentional expression of beauty, such as the gait of a runway model [44], although the strategy for expressing attractiveness in gait remains unclear. Thus, we investigated the biomechanical basis of how women express their attractiveness in walking [39].

We recruited professional fashion models (N = 7; 42.4 ± 7.0 years; 170.6 ± 3.7 cm; 55.6 ± 3.4 kg) and nonmodels (N = 10; 34.0 ± 7.2 years; 162.0 ± 5.4 cm; 54.7 ± 7.7 kg) and had them walk barefoot or in heels on a treadmill at a fixed speed of 1.0 m/s. In each footwear condition, the participants walked under two types of instructions: (1) walking as casually as possible, in the normal condition, and (2) walking as attractively as possible, in the attractive-conscious condition. Using a motion capture system, the 3D positions of the anatomical feature points of the entire body of the walker were recorded, and then we used these in subsequent offline analyses to calculate the 3D joint angles for 13 joints throughout the body. The most challenging part of this study (i.e., [39]) was selecting gait parameters to capture the expression of gait attractiveness. From the Laban movement analysis [7,8], the performer’s intention is expressed by the speed and energy of the movement, the use of space exhibited, and the movement’s geometric alignment (as shown in the silhouette). Because a gait pattern in a straight line has a limited degree of spatial freedom, this study focused on the kinetic energy and silhouette of each joint and compared them between conditions.

**Figure 1:**
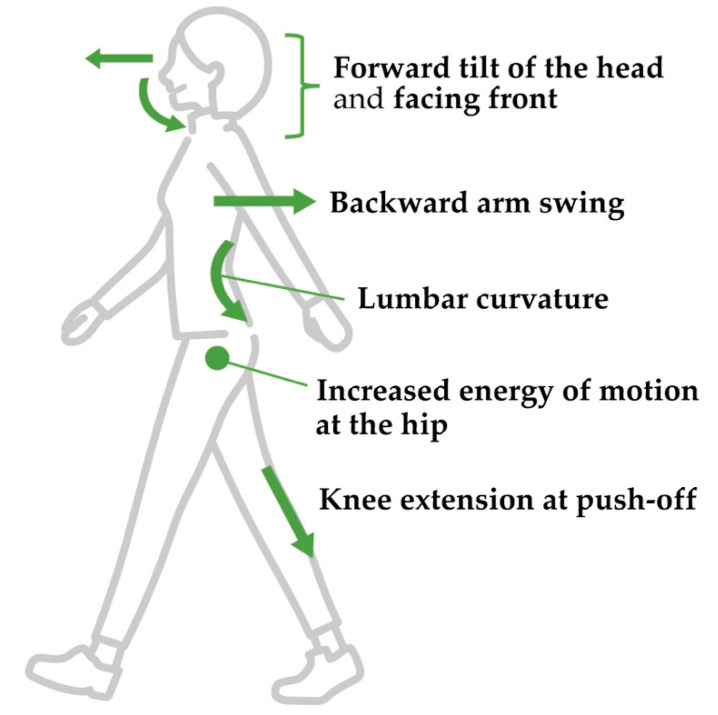
Five strategies for women to have an attractive gait.

The results suggest that female gait attractiveness is expressed through the following five strategies (Figure 1): increased hip joint energy immediately following heel contact, increased trunk curvature in the side view, head tilted and facing frontward, knee extension during push-off (i.e., before and after toe-off), and upper arm take-back. Following previous research on the characteristics of physical attractiveness, these strategies may have the function of expressing femininity and the health of walkers. For example, trunk curvature could embody the silhouette of the body in relation to reproductive function [30,31,45]. In addition, increased hip joint energy and drawing back of the upper arm have the effect of emphasizing the chest and buttocks [46]. The knee extension during push-off is associated with walking capacity, related to the muscle strength of the knee extension [47,48], which expresses the health of the walker. It should be noted that this study revealed a relationship between head silhouette and female gait attractiveness. The possible interpretations of this result are as follows: the change in head silhouette is a byproduct of the change in the alignment of the whole body, or, alternatively, the head silhouette conveys the defensive attitude of the walker [22] or emotion [19,21], which then leads observers to perceive attractiveness. The function of the head silhouette in attractiveness requires further investigation and is discussed in Section 3.2. This study showed that movements expressing health and reproductive function may be nonverbal elements inducing the perception of attractiveness (at least, performers adopt these elements as a strategy for the expression of physical attractiveness). The walking on a constant-speed treadmill captured in this study (differing in part from the gait kinematics of overground walking [49,50,51]) may affect the attractiveness ratings (which are influenced by walking speed [52,53]), so further research is needed to capture the attractiveness of gaits in more everyday environments.

### 2.2. Differences Between Fashion Models and Nonmodels: What Is the Attractive-Conscious Gait Generated by Nonmodels?

**Figure 2:**
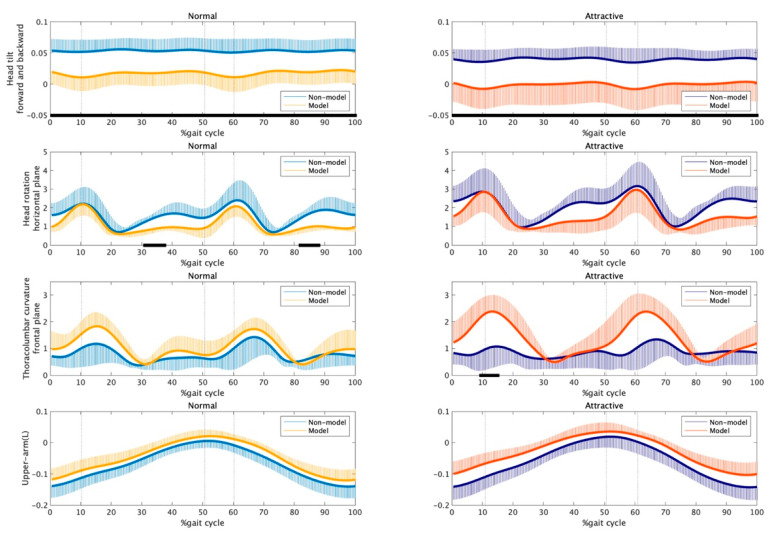
A between-group comparison in silhouette-related gait parameters: The values in the first (**left**) and second (**right**) rows show a comparison between groups in the normal and attractive-conscious conditions, respectively. The yellow and orange plots represent the model, and the blue and dark blue plots represent the nonmodel. The black bars on the horizontal axis indicate time points showing statistically significant group differences. The horizontal axis shows one stride beginning with a right-heel contact, followed by a left-toe-off (approximately 10.3%), a left-heel contact (approximately 50.7%), and a right-toe-off (approximately 60.3%), ending with a subsequent right-heel contact. Vertical axis: A larger value of each parameter (i.e., first to the fourth rows) represents a further backward head tilt, smaller head rotation in the horizontal plane (that is, facing the front), larger hip twist from the front view, and more backward arm swing, respectively.

**Figure 3:**
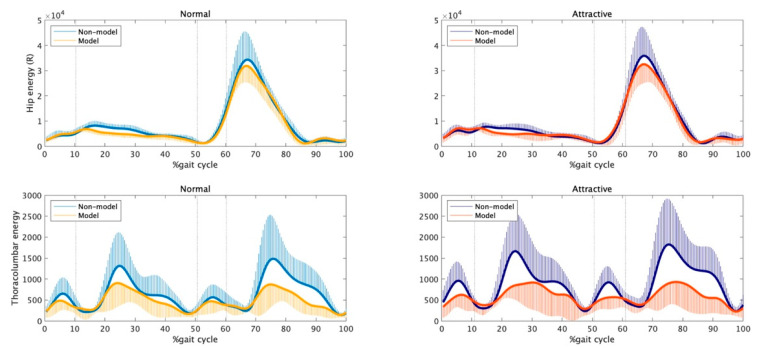
Between-group comparison of energy-related gait parameters: The values for the first (**left**) and second (**right**) rows present a comparison between groups in the normal and attractive-conscious conditions, respectively. The yellow and orange lines represent the models, and the blue and dark blue lines represent the nonmodels. The black bars on the horizontal axis present the time points using statistically significant group differences.

How did the nonmodels generate attractive gait in our study [39]? If seeking to reproduce the gait of fashion models in a neural representation, the expression strategies of attractiveness for both models and nonmodels should be similar. The focus of our study was the strategy for expressing attractiveness, and we did not examine statistical differences between the groups. Therefore, here, we additionally compared the models and nonmodels using statistical parametric mapping (SPM) [54,55]. Figure 2 and Figure 3 present time series data (% gait cycle) for the gait parameters (silhouette-related and energy-related parameters, respectively) for each group (models: blue and dark blue plots; nonmodels: yellow and orange plots) and condition (the left and right rows represent the normal and attractive-conscious conditions, respectively). The SPM{t} function using alpha = 0.05 was calculated with the SPM1D MATLAB package (http://www.spm1d.org (accessed on 15 October 2024)), and the black bars on the horizontal axis show time points with statistically significant group differences. As a result, the group differences in body silhouette in walking were observed in head tilt and rotation, as well as thoracolumbar curvature in the frontal plane (Figure 2). In other words, relative to nonmodels, models tilted their heads more forward (in both the normal and attractive-conscious conditions: 0–100% of the gait cycle), rotated their heads horizontally while pulling back their shoulders (normal condition: 31–37.5% and 82–88% of the gait cycle, with a similar tendency in the attractive-conscious condition), and with increased hip twisting when seen from the front (attractive-conscious condition: 9.5–15% of the gait cycle, around 60–65%, and a normal condition with a similar tendency). In addition, while no statistically significant difference was seen, the models tended to take their upper arms back more than the nonmodels. No statistically significant group differences were found for energy-related gait parameters (Figure 3), but the models tended to exhibit smaller hip and thoracolumbar joint energies in the double-leg support phase (around 15–35% and 65–75% for the hip and around 20–40% and 70–90% of the gait cycle).

The results above suggest that certain strategies that women adopt when expressing attractiveness go against the characteristics of the gaits of the fashion models. For example, the kinetic energy of the hip and thoracolumbar joints increased in both groups when they expressed attractiveness, but when the groups were compared, little kinetic energy was a characteristic of the model’s gait (Figure 3). In addition, the horizontal rotation of the head is smaller in the attractive-conscious condition, i.e., the head gazes straight ahead, but the horizontal rotation of the models’ heads is greater than that of the nonmodels’ heads. Of course, other attractiveness-expression strategies were consistent with the model’s gait. In the attractive-conscious condition, the woman tilts her head forward, forming a gait characteristic of fashion models. Likewise, the strategy of twisting the thoracolumbar joints on the frontal plane and taking back the upper arms is also emphasized in the models’ walks. What features were seen in the attractive-conscious gait of the nonmodels? If they were seeking to reproduce fashion models’ gait as a neural representation, the attractiveness-oriented gait of the nonmodel should go in the direction of closing the gap with the models’ gait. However, both models and nonmodels expressed patterns that were partially opposite to the strategies that are learned and embodied by models in their gait.

There may be a qualitative difference here between the expression of biological attractiveness and sociocultural attractiveness. The gait of a fashion model has as its purpose and function the showcasing of the attractiveness of clothes in the fashion industry, and the garments and styles change with the times [56]. In recent years, the tendency for models to cross their legs (employing the so-called cat walk) in their fashion display walks has decreased, and this change in gait appears to be due to the less gendered nature of clothing in a more gender-neutral or gender-fluid environment [56]. In addition, fashion model gaits are characterized by a relaxation of the upper body and a lack of excessive movement [57]. Our results can be interpreted as follows: A model’s gait is influenced by a sociocultural environment exhibiting genderlessness, with less crossing of the legs, resulting in less hip joint energy. In addition, the kinetic energy of the thoracolumbar joints is also reduced in the relaxation of the upper body, with the prevention of excessive chest movement. The increase in horizontal head rotation is a matter of further investigation, but it reflects a strategy of attracting the audience’s attention through increasing head movement [56]. Conversely, when increasing the attractiveness of their own gait, without reference to the fashion industry, women tend to increase their biological attractiveness by increasing the energy expressed in the hip and thoracolumbar joints, which could go against genderlessness. The function of the attractiveness expressed by head movements, such as facing front and having a forward tilt, needs to be clarified, but taking into account that the head silhouette can be a nonverbal message, reflecting a defensive posture [22] or emotion [19,21], a strategy to induce the perception of attractiveness could involve expressions of psychological states in one’s gait. Thus, it may be that an attractive-conscious gait is not simply a reproduction of a model’s gait but could be a biological adaptation emphasizing gender.

### 2.3. Was the Strategy of Expressing Attractiveness Successful?

Do observers perceive the attractiveness that is expressed by pedestrians? A total of 60 subjects (30 women, aged 38.50 ± 13.26 years; and 30 men, aged 40.70 ± 10.59 years) rated the attractiveness of all gaits on a 7-point scale (1: very ugly, 7: very attractive). In the barefoot condition, the attractiveness scores for the nonmodel gaits were 3.09 ± 0.53 in the normal condition and 3.50 ± 0.43 in the attractive-conscious condition, while the attractiveness scores of the models’ gaits were 3.77 ± 0.44 in the normal condition and 4.29 ± 0.64 in the attractive-conscious condition. After the normality and homogeneity of variance in the data were checked, a 2-way ANOVA was performed, and the results show a main effect of condition (F(1,30) = 6.8, *p* = 0.014, η^2^ = 0.13) and group (F(1,30) = 17.2, *p* < 0.001, η^2^ = 0.32), with no interaction. The same analysis was performed for the heel condition: the attractiveness scores for the nonmodels’ gaits were 3.49 ± 0.75 in the normal condition and 4.20 ± 0.64 in the attractive-conscious condition, while the attractiveness scores of the models’ gaits were 4.85 ± 0.69 in the normal condition and 5.41 ± 0.48 in the attractive-conscious condition. A 2-way ANOVA showed a main effect of condition (F(1,30) = 7.74, *p* = 0.0093, η^2^ = 0.11) and group (F(1,30) = 31.3, *p* < 0.001, η^2^ = 0.45), showing no interaction. These results indicate that, in both footwear conditions, the attractive-conscious gait was rated as significantly more attractive than the normal condition, where the models’ gaits were rated as more attractive than the nonmodels’ gait. The great social value of this result is that others’ attractiveness ratings are not only influenced by body shape and appearance, which are innate and unchanging or require significant time to change, but also by physical movements that can be instantly adjusted at will by the performer. Saito et al. [58] found that the tilted pelvic posture in gait affects the attractiveness evaluation, regardless of one’s figure. These facts may help steer individuals away from the ideal of thinness as promulgated in the mass media and social media [35], with its resulting unhealthy eating behaviors [59].

## 3. Attractiveness Evaluation Model of Female Gait

### 3.1. Statistical Models for Estimating Gait Attractiveness

Section 2 shows that women can change their gait to express attractiveness, and this makes observers perceive them to be more attractive. What causal relationship is there between gait parameters and attractiveness evaluation? D’Argenio et al. [60] investigated the causal relationship between body dynamics and femininity and attractiveness, revealing that body poses that are less dynamic are preferred for the female body, and that such poses are linked to attractiveness through femininity. However, they did not note the movement elements that affect the evaluation of the visual impression or their function in terms of nonverbal messages. We conducted a study to examine the function of gait parameters in attractiveness evaluation by clarifying the causal relationship between a range of gait parameters and impression scores (i.e., attractiveness and femininity) [40]. We created statistical models to explain gait attractiveness and femininity with the use of gait parameters. To construct the models, we performed structural equation modeling (SEM), which has the advantage of combining measurement models and structural (i.e., regression) models into one overarching model that can optimally handle measurement errors in predictors [61,62].

A 30 s gait animation was created using motion capture data obtained in Section 2.1. In all of the animations, the angle of presentation was rotated 180 degrees at a constant speed for 30 s to observe the walkers from the front and from the side. We recruited 60 subjects (30 women, aged 38.50 ± 13.26 years, and 30 men, aged 40.70 ± 10.59 years), and each subject watched all of the animations, rating the attractiveness and femininity of each animation on a 7-point Likert scale (as above). To obtain explanatory variables for SEM, we first performed a correlation analysis between the impression scores and the walkers’ parameters, including gait parameters and physical characteristics. The walkers’ parameters were as follows: height, weight, BMI, waist-to-hip ratio, lumbar curvature, upper arm pull-back, forward head tilt, horizontal rotation of the head, stride CV, cadence, clearance, symmetry, knee extension, and toe-off angle. Next, we used the parameters of the walkers with a moderate or higher correlation with the impression score as explanatory variables to construct statistical models using SEM. Because it is possible for observers to distinguish between barefoot and heel walking, SEM was constructed for the footwear conditions separately.

**Figure 4:**
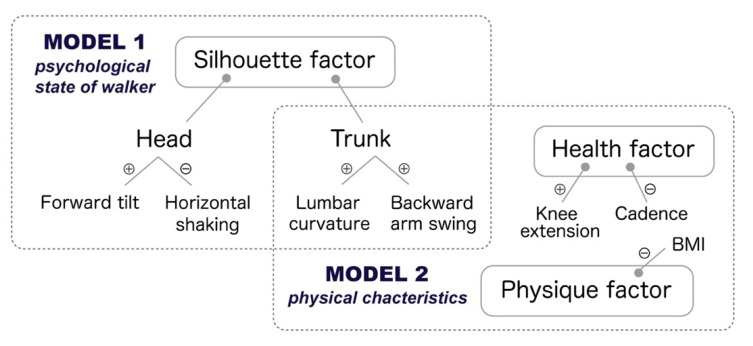
An overview of the walkers’ elements affecting the observers’ attractiveness perception. Models 1 and 2 indicate the evaluation criteria for attractiveness (silhouette-related and health–physique-related standards, respectively) in the two models that were constructed in our previous study [40]. The positive and negative signs indicate whether larger or smaller values lead to higher attractiveness ratings.

From the correlation analysis, BMI, lumbar curve (barefoot only), upper arm take-back, head forward tilt, horizontal rotation of the head (heels only), cadence, clearance (heels only), knee extension, and toe-off angle (barefoot only) were moderately correlated with impression scores. Using SEM with these parameters as explanatory variables, two models were constructed for each footwear condition: one model consisting of silhouette elements of the head and trunk (Model 1), and a model consisting of body shape, trunk silhouette, and health elements (Model 2). We attempted to merge Models 1 and 2, but they diverged, which is likely because they were independent. Figure 4 forms an overview of the walkers’ elements, which affected the observers’ perception of attractiveness, based on the SEM models constructed in [40]. The functions of Models 1 and 2 are discussed in Section 3.2. Because it is possible to predict target values based on SEM models [63], this study likewise made it possible to estimate gait attractiveness using gait parameters and artificially tune gait attractiveness in the generation of biological motion.

### 3.2. Function of Gait Attractiveness in Evolutionary Psychological and Sociocultural Contexts

Model 2 (Figure 4) is composed of the elements of health-related gait parameters, body shape, and trunk silhouette in relation to femininity, which are the criteria reflecting the walker’s physical characteristics. In support of previous studies [30,31,58], it was found that the lumbar curvature (i.e., the anterior pelvic tilt) promotes the observer’s perception of attractiveness in walking movements. The criterion of the knee extension, which decreases with age, is also consistent with the explanation of attractiveness according to evolutionary psychology, which states that the perception of attractiveness is a psychological process that detects an individual’s health (e.g., [32,33]). Our findings suggest that observers sense information related to evolutionary adaptability using visual information of the movements of the body, linking it to the perception of attractiveness. However, other evaluation criteria went against the perception of health. BMI is an index of weight, where a normal weight is between 20 and 24.9 kg/m^2^ [64]. The BMI of the walkers in this study was 20.01 ± 1.89 (minimum of 17.02 and maximum of 24.07), within the range of underweight to normal weight. A negative correlation was observed between the BMI and attractiveness score. This is consistent with previous research, which shows that women having BMIs lower than the healthy standard are preferred [28], which may reflect a feature of physical appearance that is shaped by the sociocultural environments [65], such as in the tendency of the mass media to promote underweight body appearance as part of the standard of beauty as a gender role norm for girls [35,66,67]. In addition, the negative correlation we found between cadence and attractiveness scores [40] is inconsistent with an association between health and attractiveness, in that lower cadences reflect a reduced ability to modulate the gait cycle duration, which can be linked to age-related diseases, such as knee osteoarthritis [68]. Why does attractiveness perception based on the physical characteristics of walkers both coincide with and contradict their health? One reason for this may be that the evaluations conducted by men and women are mixed. From the perspective of evolutionary psychology, males’ evaluations of female attractiveness should have qualitative differences from females’ evaluations of female attractiveness. We discuss this point in Section 4.

Model 1 (Figure 4) is based on the silhouette of the trunk and head. To the best of our knowledge, the relationship and function between head alignment and attractiveness is unclear. Head alignment is linked to emotional expression and interpersonal attitudes. For example, sadness [19,21] and a defensive posture [22] are expressed in a tilt of the head. In addition, the relationship between the horizontal rotation of the head is a negative correlation with the attractiveness score, which suggests that a silhouette with the head tilted forward and facing front in walking may convey a message of low openness and conservatism toward others, and this psychological state may be perceived as favorable. This is consistent with a preference for a static pose in female bodies [60]. Thus, Model 1 could form a criterion to judge the walker’s emotions and interpersonal attitudes. Where first impressions are being formed in romantic relationships, women tend to communicate their approachability in nonverbal behaviors, such as tilting their head, shrugging their shoulders, looking down, looking to the side, frowning, holding hands, hugging, patting, having their hands in front of their bodies, and smoothing their hair [69]. Therefore, head movements are important for conveying women’s nonverbal immediacy. The function and meaning of head movements in women’s nonverbal immediacy must be further examined. In summary, it was suggested that the attractiveness of the female gait is determined by two criteria (Figure 4): the physical state of the walker, i.e., both evolutionarily adaptive and sociocultural criteria (Model 2), and the walker’s interpersonal attitude (Model 1).

## 4. Perception of Gait Attractiveness

### 4.1. Gaze Behavior Associated with Gait Attractiveness Evaluation and Its Sex Differences

Section 3 identifies the criteria according to which observers evaluate the female gait attractiveness. What gaze behavior gathers information on gait and leads to the perception of attractiveness and positive evaluation? Gender differences appear in the perception of physical attractiveness. For example, in the observation of both men’s and women’s body images, male observers pay attention sooner and for longer to women’s chests, while female observers pay attention sooner to men’s legs [70]. Moreover, men pay more attention to female breasts and heads than women do [71]. Pazhoohi et al. [72] also show that there are sex differences in terms of the prominence of the shoulder-to-hip ratio (SHR), where men paid more attention to SHR. Here, two forces of sexual selection exist: intersexual (i.e., mate choice) and intrasexual (i.e., the sexual competition between individuals of the same sex) selection [73]. In human sexual selection, physical attractiveness is an important criterion in mate selection [74], and women compete with the same sex through attracting men, while men tend to compete with the same sex through physical strength [75]. We thus investigated what bodily cues are used to assess female gait attractiveness, focusing not only on men’s evaluation of women, which could affect mate selection, but also on women’s evaluation of their potential competitors [41]. In addition, when an observer perceives attractiveness, their attention interacts with their preference of bodily appearance [76]. We thus investigated the relationship between gait attractiveness and gaze behavior, in addition to sex differences.

We analyzed the gaze behavior of 33 (17 women; 38.06 ± 14.21 years, and 16 men; 42.25 ± 10.32 years) of the 60 participants in the impression evaluation experiment described in Section 3. We analyzed their gaze-tracking data using eye-tracking software (Tobii Pro Lab, Screen Edition, version 1.181) and a Tobii Pro Nano screen-based eye-tracking camera (Tobii, Danderyd, Sweden) attached to the bottom of the computer monitor. We set five AOIs (head, trunk, hip, leg, and others) for each walking animation in the software, and we calculated the fixation rate for each AOI and each participant. The observers’ gaze area could have been influenced by their sex and preferences (i.e., for gait attractiveness). Therefore, we compared fixation rates according to the four factors of sex, preference (i.e., top vs. bottom 5 animations in attractiveness ranking among 68 animations), phase (phase 0 is the first 1.0 s from the start of the animation, and phases 1–4 are each a period of 7.5 s, totaling 30 s), and AOI (i.e., head, trunk, hip, leg, and others). We also constructed models that link gait parameters and the attractiveness scores for each observer’s sex by using SEM and qualitatively compared standardized estimates.

A sex difference was found in visual attention in the fixation rates of the trunk and leg. Male observers were highly fixed on the trunk, while female observers tended to shift their attention from the trunk to the legs, especially in the observation of high-preference gait animations. In addition, the models constructed by SEM for each sex showed a tendency for male observers to place a greater weight on the walkers’ trunk silhouette, while female observers prioritized parameters requiring whole-body observation. In human sexual selection, women’s physical attractiveness is an important criterion for men’s mate selection [74,77,78,79], and women compete with the same sex by attracting men [75]. We found that in the evaluation of female walkers’ attractiveness, men place importance on the gait silhouette of the female trunk, which supports the idea that the reproductive regions of the female body are important for men in mate choices. Meanwhile, female observers tended to highlight BMI, knee extension, cadence, and head silhouette, requiring full-body observation when evaluating female gait attractiveness. Considering that the preferred women’s BMI and fat mass values are below the healthy standard [28] and that mass media uses underweight bodies as icons of beauty and gender norms for girls [35,66,67], women may consider other women’s attractiveness in relation to the dominant beauty standards of their sociocultural environment [65]. Further, taking into account that head silhouettes can function as nonverbal messages that reflect a walker’s interpersonal attitude [22] and emotions [19,21], the walker’s psychological state is a potential factor used in evaluating the attractiveness of potential competitors.

### 4.2. Visual Information Processing of Gait Attractiveness

The central nervous system processes visual information in a hierarchical and parallel manner [80,81,82]. Sequential hierarchy is divided into three stages [83]: an early vision that extracts simple elements such as color, shape, motion, and position [84,85]; intermediate vision, which groups elements into coherent entities [86,87,88,89]; and late vision, which selects areas of coherence for the direction of attention and recalling memories that allow us to recognize and attach meaning to objects [80,90]. According to neuroaesthetics [91,92], an observer’s perception of an aesthetic object includes visual information processing, impression evaluation, and emotional reaction to the object. Each of these processes can be further deconstructed as follows: Once the basic visual elements of the object have been received, this information is collated in higher-level cognitive and attentional processes to form an impression and a final determination of aesthetic evaluation. This principle applies not only to visual aesthetics but also to music, dance, literature, and aesthetic judgments of the body [92]. In the evaluation of gait attractiveness, the motion of walking as a stimulus was initially deconstructed into visual features and sorted and processed. Then, higher-level cognitive and attentional processes are used to evaluate walkers’ gaits attractiveness.

**Figure 5:**
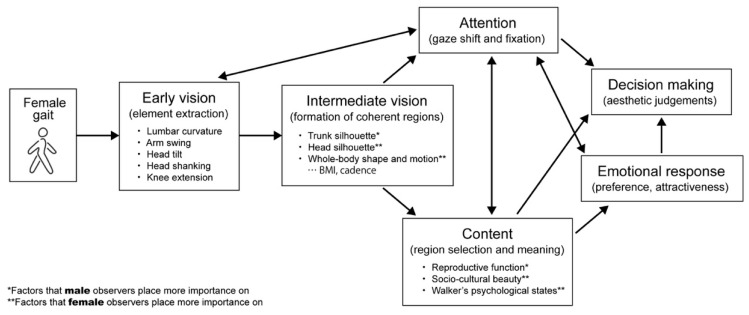
Visual information processing for the attractiveness perception and evaluation of female gaits. The arrows represent the flow of visual processing.

Drawing on information processing in neuroaesthetics [92], we created a model that showed the visual information processing of the attractiveness of female gaits (Figure 5). When walking motion is input as visual information, gait parameters such as lumbar curvature and head tilt are first extracted in early vision, and then they are recognized as a whole in intermediate vision. Here, male observers emphasize the trunk silhouette, while female observers emphasize the silhouette and movement of the head and whole body, and attention is induced in such sex-dependent vision. Based on this information, men and women attach meanings that are related to reproductive function, sociocultural standards of beauty, and the psychological state of the walker, respectively. In this way, the extraction of visual elements, attention, and meaning (i.e., context) interact to evoke the emotional responses of attractiveness and the final decision making of aesthetic judgment. Because attention and emotional response interact (i.e., they induce each other) [76], a reciprocal path appears between them. To the best of our knowledge, this is the first attempt to build an information processing model for gait attractiveness. It not only deepens our understanding of the central perceptual processes of physical attractiveness but also provides a deeper descriptive texture to bodily aesthetics (i.e., the psychological nature of the reward of the attractive experience). However, our study features limitations, such as the limitation of the visual stimuli to the female gait and the observers being limited to individuals of Japanese culture in a small sample. Thus, further verification is needed.

## 5. Conclusions

In this paper, we deepened our understanding of the meaning and function of walking in terms of nonverbal information regarding our previous study of the expression and perception of gait attractiveness in a comprehensive literature review. In Section 2, we focused on gait patterns used by women to express attractiveness and introduced five strategies of increase in hip joint energy immediately after heel contact, increase in trunk curvature in the side view, tilt and front facing of the head, knee extension during push-off (i.e., before and after toe-off), and upper arm drawback. Additional analysis of the differences between models and nonmodels showed the following qualitative differences between the expression of biological attractiveness and cultural attractiveness: In the models’ gait, influenced by the sociocultural environment, leg crossing is reduced, which reduces the energy of the hip joint. In addition, the kinetic energy of the thoracolumbar joint is reduced to prevent the excessive movement of the upper body. These features are thought to be due to the trend toward genderlessness in the fashion industry. However, when trying to increase the attractiveness of their gait, women, including fashion models, increase the energy of their hip and thoracolumbar joints, which is in tension with genderlessness. Thus, the generation of attractive gait in nonmodels may not be a reproduction of the gait of fashion models but a biological adaptation to emphasize gender.

In Section 3 and Section 4, we discussed the link between the gait parameters and evaluation of attractiveness, the visual information processing of the gait in observers, and gait attractiveness in relation to the evolutionary psychological and sociocultural perspectives. There are not only criteria for gait attractiveness consistent with the reproductive function and health of walkers but also sociocultural criteria that contradict them. Male observers tend to place more importance on the former criteria when evaluating female gait attractiveness as intersexual selection, while female observers tend to assign more importance to the latter when evaluating the attractiveness of their competitors in intrasexual selection. From these results, we proposed a visual information processing model for the attractiveness perception and evaluation of female gaits. This forms a major step forward in the neuroaesthetics of the beauty of the human body. The generation of biological motion for walking is widely used in the robot and AI industries, and the review in this paper provides a useful psychological basis for industrial implementation. In further studies, we plan to investigate the function of attractiveness of human movements and behavior, including social factors like clothing and context.
